# Crystal structure of *p*-toluene­sulfonyl­methyl isocyanide

**DOI:** 10.1107/S2056989015008816

**Published:** 2015-05-20

**Authors:** Huma Bano, Sammer Yousuf

**Affiliations:** a H. E. J. Research Institute of Chemistry, International Center for Chemical and Biological Sciences, University of Karachi, Karachi 75270, Pakistan

**Keywords:** crystal structure, isocyanide derivative, hydrogen bonding

## Abstract

The mol­ecule of the commercially available title compound, C_9_H_9_NO_2_S, has crystallographically imposed mirror symmetry, the mirror plane passing through the isocyanide group and the *para*-C atoms, the methyl C atom and the S atom of the methyl 4-tolyl sulfone moiety. In the crystal, C—H⋯O hydrogen-bond inter­actions link the mol­ecules into chains running parallel to the *b* axis.

## Related literature   

The title compound is an isocyanide derivative of methyl 4-tolyl sulfone (Ye, 2007 ▸), an important reaction inter­mediate obtained during the synthesis of mesotrione, a well known herbicide (Smith *et al.*, 2008 ▸).
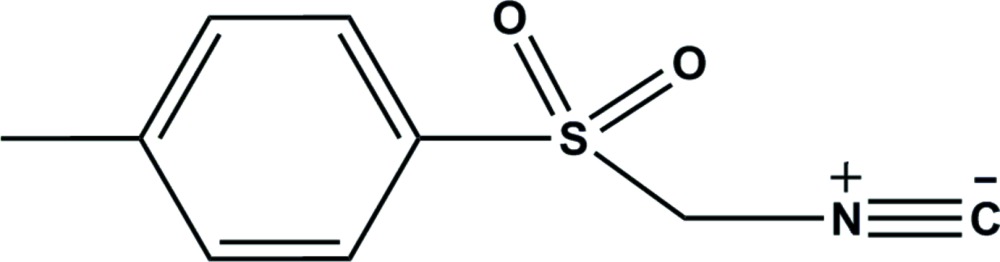


## Experimental   

### Crystal data  

C_9_H_9_NO_2_S
*M* *_r_* = 195.23
Orthorhombic, 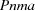

*a* = 22.342 (5) Å
*b* = 8.881 (2) Å
*c* = 4.8462 (12) Å
*V* = 961.6 (4) Å^3^
*Z* = 4
Mo *K*α radiation
μ = 0.30 mm^−1^
*T* = 273 K
0.49 × 0.32 × 0.15 mm

### Data collection  

Bruker SMART APEX CCD area-detector diffractometer
Absorption correction: multi-scan (*SADABS*; Bruker, 2000 ▸) *T*_min_ = 0.864, *T*_max_ = 0.961
5160 measured reflections
955 independent reflections
733 reflections with *I* > 2σ(*I*)
*R*_int_ = 0.044

### Refinement  

*R*[*F* ^2^ > 2σ(*F* ^2^)] = 0.047
*wR*(*F* ^2^) = 0.119
*S* = 1.11
955 reflections
77 parameters
1 restraint
H atoms treated by a mixture of independent and constrained refinement
Δρ_max_ = 0.39 e Å^−3^
Δρ_min_ = −0.19 e Å^−3^

### 

Data collection: *SMART* (Bruker, 2000 ▸); cell refinement: *SAINT* (Bruker, 2000 ▸); data reduction: *SAINT*; program(s) used to solve structure: *SHELXS97* (Sheldrick, 2008 ▸); program(s) used to refine structure: *SHELXL97* (Sheldrick, 2008 ▸); molecular graphics: *SHELXTL* (Sheldrick, 2008 ▸); software used to prepare material for publication: *SHELXTL*, *PARST* (Nardelli, 1995 ▸) and *PLATON* (Spek, 2009 ▸).

## Supplementary Material

Crystal structure: contains datablock(s) global, I. DOI: 10.1107/S2056989015008816/rz5158sup1.cif

Structure factors: contains datablock(s) I. DOI: 10.1107/S2056989015008816/rz5158Isup2.hkl

Supporting information file. DOI: 10.1107/S2056989015008816/rz5158Isup3.cml

**Fig. 1.**
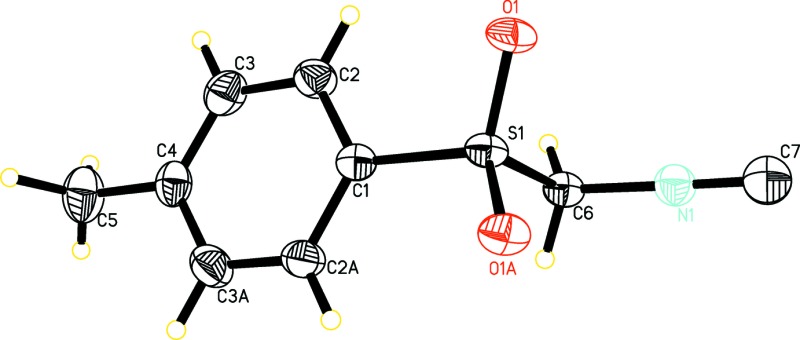
The molecular structure of title compound with displacement ellipsoids drawn at the 30% probability level.

**Fig. 2.**
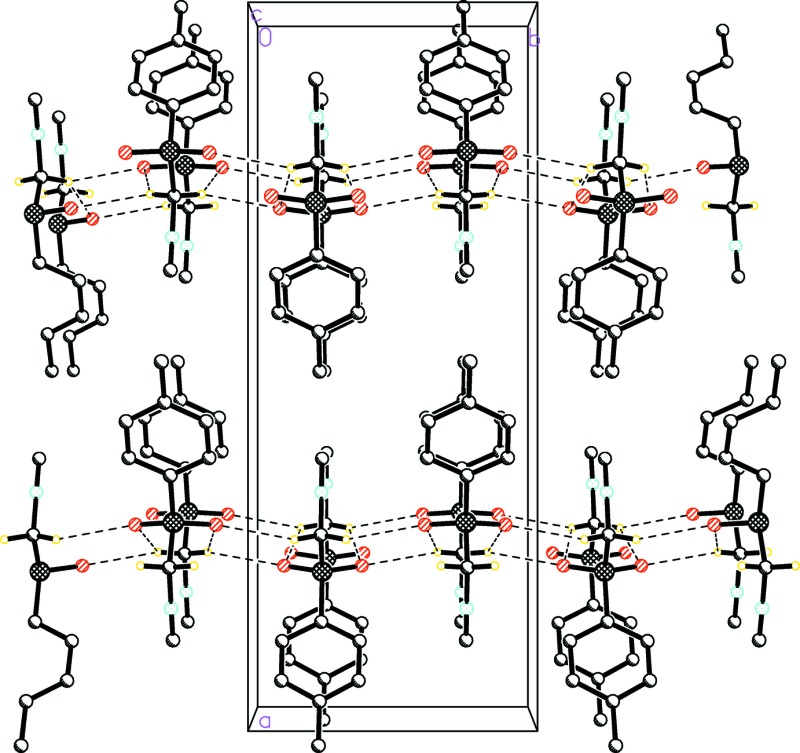
Crystal packing of the title compound, showing the formation of chains parallel to the b axis via C—H···O hydrogen bonds (dashed lines). H atoms not involved in hydrogen bonding are omitted.

. DOI: 10.1107/S2056989015008816/rz5158fig1.tif
The mol­ecular structure of title compound with displacement ellipsoids drawn at the 30% probability level.

b via . DOI: 10.1107/S2056989015008816/rz5158fig2.tif
Crystal packing of the title compound, showing the formation of chains parallel to the *b* axis *via* C—H⋯O hydrogen bonds (dashed lines). H atoms not involved in hydrogen bonding are omitted.

CCDC reference: 1063415

Additional supporting information:  crystallographic information; 3D view; checkCIF report

## Figures and Tables

**Table 1:** Hydrogen-bond geometry (, )

*D*H*A*	*D*H	H*A*	*D* *A*	*D*H*A*
C6H6*A*O1^i^	0.97	2.47	3.2519(18)	138
C6H6*A*O1^ii^	0.97	2.54	3.296(4)	135
C6H6*B*O1^iii^	0.97	2.54	3.296(4)	135
C6H6*B*O1^iv^	0.97	2.47	3.2519(18)	138

Symmetry codes: (i) 
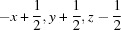
; (ii) 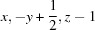; (iii) 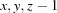; (iv) 
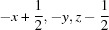
.

## supplementary crystallographic information

### S1. Comment

The title compound is an isocyanide derivative of the previously reported compound methyl 4-tolyl sulfone (Ye, 2007), an important reaction intermediate obtained during the synthesis of mesotrione, a well known herbicide (Smith *et al.*, 2008). The compound was crystallized as part of our ongoing research project involving the study of the crystal structures and enzyme inhibition abilities of commercially available molecular libraries. The molecule has crystallographically imposed mirror symmetry, atoms C1, C4–C7, N1, S1 lying on the mirror plane (Fig. 1). The least-square mean line through C6, N1 and C7 forms an angle of 79.4 (3)° with the normal to the plane of the benzene ring. The crystal structure is stabilized by C6—H6A···O1, and C6—H6B···O1 intermolecular hydrogen interactions that link the molecules to form chains running parallel to the *b* axis (Fig. 2).

### S2. Experimental

The title compound is a commercially available Sigma-Aldrich product. Colourless single crystals suitable for X-ray analysis were obtained from slow evaporation of a methanol solution at room temperature.

### S3. Refinement

Aromatic and methylene H atoms were positioned geometrically and constrained to ride on their parent atoms, with C—H = 0.93-0.97 Å, and with *U*_iso_(H)= 1.2*U*_eq_(C). The methyl H5A atom lying on a mirror plane was located in a difference Fourier map and refined isotropically, with the C5–H5A bond length constrained to be 1.1 (1) Å.

### Crystal data

**Table:**

C_9_H_9_NO_2_S	*F*(000) = 408
*M**_r_* = 195.23	*D*_x_ = 1.349 Mg m^−^^3^
Orthorhombic, *P**n**m**a*	Mo *K*α radiation, λ = 0.71073 Å
Hall symbol: -P 2ac 2n	Cell parameters from 830 reflections
*a* = 22.342 (5) Å	θ = 2.9–22.3°
*b* = 8.881 (2) Å	µ = 0.30 mm^−^^1^
*c* = 4.8462 (12) Å	*T* = 273 K
*V* = 961.6 (4) Å^3^	Plate, colourless
*Z* = 4	0.49 × 0.32 × 0.15 mm

### Data collection

**Table:**

Bruker SMART APEX CCD area-detector diffractometer	955 independent reflections
Radiation source: fine-focus sealed tube	733 reflections with *I* > 2σ(*I*)
Graphite monochromator	*R*_int_ = 0.044
ω scan	θ_max_ = 25.5°, θ_min_ = 1.8°
Absorption correction: multi-scan (*SADABS*; Bruker, 2000)	*h* = −27→25
*T*_min_ = 0.864, *T*_max_ = 0.961	*k* = −10→10
5160 measured reflections	*l* = −5→5

### Refinement

**Table:**

Refinement on *F*^2^	Primary atom site location: structure-invariant direct methods
Least-squares matrix: full	Secondary atom site location: difference Fourier map
*R*[*F*^2^ > 2σ(*F*^2^)] = 0.047	Hydrogen site location: inferred from neighbouring sites
*wR*(*F*^2^) = 0.119	H atoms treated by a mixture of independent and constrained refinement
*S* = 1.11	*w* = 1/[σ^2^(*F*_o_^2^) + (0.0557*P*)^2^ + 0.1844*P*] where *P* = (*F*_o_^2^ + 2*F*_c_^2^)/3
955 reflections	(Δ/σ)_max_ < 0.001
77 parameters	Δρ_max_ = 0.39 e Å^−^^3^
1 restraint	Δρ_min_ = −0.19 e Å^−^^3^

### Special details

**Table:**

Geometry. All e.s.d.'s (except the e.s.d. in the dihedral angle between two l.s. planes) are estimated using the full covariance matrix. The cell e.s.d.'s are taken into account individually in the estimation of e.s.d.'s in distances, angles and torsion angles; correlations between e.s.d.'s in cell parameters are only used when they are defined by crystal symmetry. An approximate (isotropic) treatment of cell e.s.d.'s is used for estimating e.s.d.'s involving l.s. planes.
Refinement. Refinement of *F*^2^ against ALL reflections. The weighted *R*-factor *wR* and goodness of fit *S* are based on *F*^2^, conventional *R*-factors *R* are based on *F*, with *F* set to zero for negative *F*^2^. The threshold expression of *F*^2^ > σ(*F*^2^) is used only for calculating *R*-factors(gt) *etc*. and is not relevant to the choice of reflections for refinement. *R*-factors based on *F*^2^ are statistically about twice as large as those based on *F*, and *R*- factors based on ALL data will be even larger.

### Fractional atomic coordinates and isotropic or equivalent isotropic displacement parameters (Å^2^)

**Table:**

	*x*	*y*	*z*	*U*_iso_*/*U*_eq_	Occ. (<1)
S1	0.28836 (4)	0.2500	0.85946 (16)	0.0354 (3)	
O1	0.28301 (8)	0.11111 (18)	1.0084 (3)	0.0462 (5)	
N1	0.17475 (16)	0.2500	0.7198 (6)	0.0483 (8)	
C1	0.35416 (16)	0.2500	0.6642 (7)	0.0379 (9)	
C2	0.37872 (13)	0.1154 (3)	0.5815 (6)	0.0488 (7)	
H2A	0.3624	0.0246	0.6398	0.059*	
C3	0.42793 (13)	0.1177 (3)	0.4109 (6)	0.0557 (8)	
H3A	0.4446	0.0268	0.3543	0.067*	
C4	0.45334 (18)	0.2500	0.3212 (7)	0.0477 (10)	
C5	0.5064 (2)	0.2500	0.1335 (11)	0.0714 (16)	
H5B	0.505 (2)	0.320 (5)	0.013 (11)	0.14 (2)*	
C6	0.23235 (16)	0.2500	0.5941 (7)	0.0383 (9)	
H6A	0.2369	0.3385	0.4788	0.046*	0.50
H6B	0.2369	0.1615	0.4788	0.046*	0.50
C7	0.1292 (2)	0.2500	0.8320 (11)	0.0707 (14)	
H5A	0.549 (2)	0.2500	0.242 (17)	0.21 (4)*	

### Atomic displacement parameters (Å^2^)

**Table:**

	*U*^11^	*U*^22^	*U*^33^	*U*^12^	*U*^13^	*U*^23^
S1	0.0508 (6)	0.0300 (5)	0.0255 (4)	0.000	0.0031 (4)	0.000
O1	0.0676 (13)	0.0360 (10)	0.0349 (10)	0.0001 (9)	0.0045 (9)	0.0096 (7)
N1	0.051 (2)	0.0469 (19)	0.0467 (18)	0.000	0.0048 (17)	0.000
C1	0.044 (2)	0.0368 (19)	0.0330 (18)	0.000	−0.0023 (16)	0.000
C2	0.0551 (18)	0.0383 (15)	0.0531 (15)	−0.0007 (13)	0.0084 (14)	−0.0045 (12)
C3	0.0549 (19)	0.0551 (18)	0.0572 (18)	0.0105 (15)	0.0053 (15)	−0.0108 (15)
C4	0.040 (2)	0.065 (3)	0.039 (2)	0.000	−0.0021 (17)	0.000
C5	0.055 (3)	0.104 (5)	0.056 (3)	0.000	0.012 (3)	0.000
C6	0.051 (2)	0.0355 (18)	0.0288 (17)	0.000	0.0018 (16)	0.000
C7	0.066 (3)	0.059 (3)	0.087 (4)	0.000	0.008 (3)	0.000

### Geometric parameters (Å, º)

**Table:**

S1—O1^i^	1.4340 (16)	C2—H2A	0.9300
S1—O1	1.4341 (16)	C3—C4	1.375 (4)
S1—C1	1.748 (4)	C3—H3A	0.9300
S1—C6	1.794 (4)	C4—C3^i^	1.375 (4)
N1—C7	1.154 (5)	C4—C5	1.494 (6)
N1—C6	1.424 (5)	C5—H5B	0.85 (5)
C1—C2^i^	1.375 (3)	C5—H5A	1.095 (10)
C1—C2	1.375 (3)	C6—H6A	0.9700
C2—C3	1.376 (4)	C6—H6B	0.9700
			
O1^i^—S1—O1	118.66 (14)	C4—C3—H3A	118.9
O1^i^—S1—C1	110.03 (9)	C2—C3—H3A	118.9
O1—S1—C1	110.03 (9)	C3—C4—C3^i^	117.3 (4)
O1^i^—S1—C6	107.61 (10)	C3—C4—C5	121.33 (18)
O1—S1—C6	107.61 (10)	C3^i^—C4—C5	121.33 (18)
C1—S1—C6	101.45 (16)	C4—C5—H5B	113 (3)
C7—N1—C6	177.2 (4)	C4—C5—H5A	114 (5)
C2^i^—C1—C2	120.8 (3)	H5B—C5—H5A	111 (4)
C2^i^—C1—S1	119.56 (18)	N1—C6—S1	108.9 (2)
C2—C1—S1	119.56 (18)	N1—C6—H6A	109.9
C1—C2—C3	118.8 (3)	S1—C6—H6A	109.9
C1—C2—H2A	120.6	N1—C6—H6B	109.9
C3—C2—H2A	120.6	S1—C6—H6B	109.9
C4—C3—C2	122.2 (3)	H6A—C6—H6B	108.3
			
O1^i^—S1—C1—C2^i^	−25.6 (3)	S1—C1—C2—C3	175.2 (2)
O1—S1—C1—C2^i^	−158.2 (2)	C1—C2—C3—C4	0.2 (5)
C6—S1—C1—C2^i^	88.1 (3)	C2—C3—C4—C3^i^	0.6 (6)
O1^i^—S1—C1—C2	158.2 (2)	C2—C3—C4—C5	−179.3 (4)
O1—S1—C1—C2	25.6 (3)	O1^i^—S1—C6—N1	−64.47 (9)
C6—S1—C1—C2	−88.1 (3)	O1—S1—C6—N1	64.47 (9)
C2^i^—C1—C2—C3	−0.9 (6)	C1—S1—C6—N1	180.0

Symmetry code: (i) *x*, −*y*+1/2, *z*.

### Hydrogen-bond geometry (Å, º)

**Table:**

*D*—H···*A*	*D*—H	H···*A*	*D*···*A*	*D*—H···*A*
C6—H6*A*···O1^ii^	0.97	2.47	3.2519 (18)	138
C6—H6*A*···O1^iii^	0.97	2.54	3.296 (4)	135
C6—H6*B*···O1^iv^	0.97	2.54	3.296 (4)	135
C6—H6*B*···O1^v^	0.97	2.47	3.2519 (18)	138

Symmetry codes: (ii) −*x*+1/2, *y*+1/2, *z*−1/2; (iii) *x*, −*y*+1/2, *z*−1; (iv) *x*, *y*, *z*−1; (v) −*x*+1/2, −*y*, *z*−1/2.
