# Supplementary figures and images for: Crystal structure of p-toluene­sulfonyl­methyl isocyanide
Source: Acta Crystallogr E Crystallogr Commun. 2015 May 20;71(Pt 6):o412. doi: 10.1107/S2056989015008816 (PMC4459310; doi:10.1107/S2056989015008816)

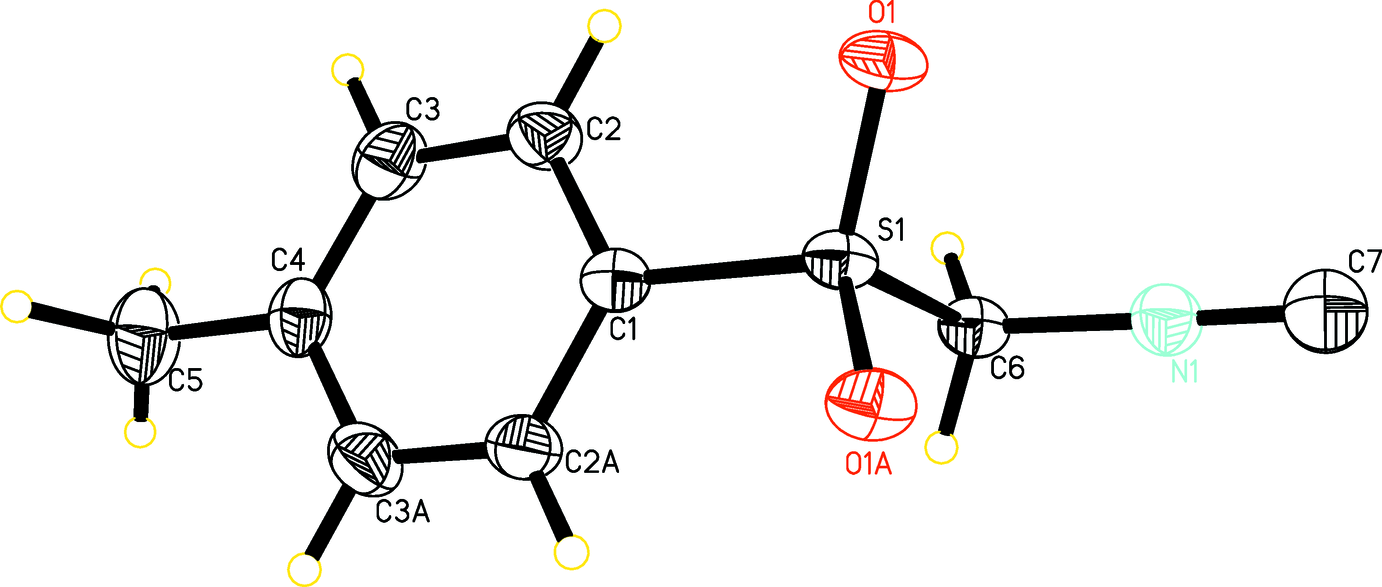

Supplement: Supplementary file 4 [file e-71-0o412-fig1.tif]

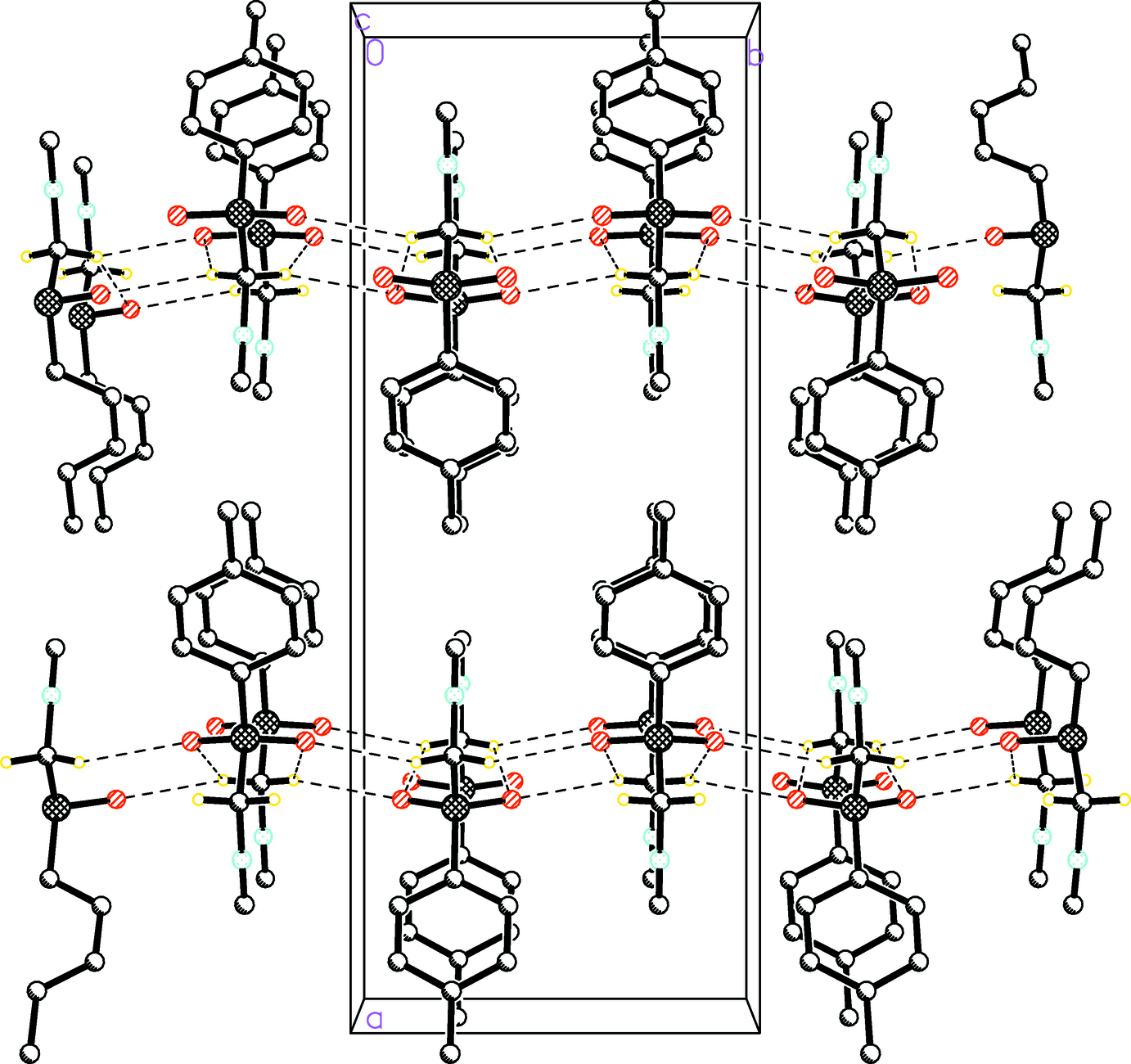

Supplement: Supplementary file 5 [file e-71-0o412-fig2.tif]
